# Tailoring Nylon 6/Acrylonitrile-Butadiene-Styrene Nanocomposites for Application against Electromagnetic Interference: Evaluation of the Mechanical, Thermal and Electrical Behavior, and the Electromagnetic Shielding Efficiency

**DOI:** 10.3390/ijms23169020

**Published:** 2022-08-12

**Authors:** Carlos Bruno Barreto Luna, Emanuel Pereira do Nascimento, Danilo Diniz Siqueira, Bluma Guenther Soares, Pankaj Agrawal, Tomás Jeferson Alves de Mélo, Edcleide Maria Araújo

**Affiliations:** 1 Academic Unit of Materials Engineering, Federal University of Campina Grande, Av. Aprígio Veloso, 882-Bodocongó, Campina Grande 58429-900, PB, Brazil; 2 Department of Metallurgic and Materials Engineering, Macromolecules Institute, Federal University of Rio de Janeiro, Rio de Janeiro 21941-598, RJ, Brazil

**Keywords:** polymeric nanocomposites, carbon nanotubes, electrical conductivity, electromagnetic shielding, electrical sector

## Abstract

Nylon 6/acrylonitrile-butadiene-styrene nanocomposites were prepared by mixing in a molten state and injection molded for application in electromagnetic interference shielding and antistatic packaging. Multi-wall carbon nanotubes (MWCNT) and maleic anhydride-grafted ABS compatibilizer were incorporated to improve the electrical conductivity and mechanical performance. The nanocomposites were characterized by oscillatory rheology, Izod impact strength, tensile strength, thermogravimetry, current-voltage measurements, shielding against electromagnetic interference, and scanning electron microscopy. The rheological behavior evidenced a severe increase in complex viscosity and storage modulus, which suggests an electrical percolation phenomenon. Adding 1 to 5 phr MWCNT into the nanocomposites produced electrical conductivities between 1.22 × 10^−6^ S/cm and 6.61 × 10^−5^ S/cm. The results make them suitable for antistatic purposes. The nanocomposite with 5 phr MWCNT showed the highest electromagnetic shielding efficiency, with a peak of –10.5 dB at 9 GHz and a value around –8.2 dB between 11 and 12 GHz. This was possibly due to the higher electrical conductivity of the 5 phr MWCNT composition. In addition, the developed nanocomposites, regardless of MWCNT content, showed tenacious behavior at room temperature. The results reveal the possibility for tailoring the properties of insulating materials for application in electrical and electromagnetic shielding. Additionally, the good mechanical and thermal properties further widen the application range.

## 1. Introduction

Nanocomposites constitute a class of materials formed by hybrids of organic and inorganic materials, wherein the inorganic nanometric phase is dispersed in a polymer matrix [1,2]. In the last decades, widespread interest in polymer nanocomposites has been motivated by their tuned mechanical, optical, electrical, and magnetic properties [3,4,5]. The preparation of nanocomposites may involve many different nanofillers, such as graphite, clay, silica, carbon nanotubes, graphene, and magnetic ferrites [6,7,8,9,10]. Of note, among the various existing engineering polymers, Nylon 6 has received special attention for use in nanocomposite development [11,12].

Nylon 6 (N) is a semicrystalline polymer with a glass transition temperature (T_g_) between 45 and 52 °C and a melting point (T_m_) around 220 °C [13]. In general, Nylon 6 has high rigidity, good abrasion resistance, high tensile strength, high fatigue strength, high service temperature, and good toughness above its T_g_. These outstanding properties justify its many applications, such as in mechanical parts, electronic components, and parts for the automotive industry [14,15,16]. Nylon 6 is considered a pseudo-ductile polymer, having high resistance to crack initiation compared with brittle polymer matrices. However, it is inherently brittle in the presence of stress concentrators, such as a notch [17]. Some works have addressed this problem by designing Nylon 6/clay nanocomposites [18,19]. The Nylon 6/clay systems showed improved tensile strength, toughness, thermal stability, flame resistance, and thermomechanical strength. Recently, researchers have focused on conductive nanofiller-based nanocomposites for antistatic packaging and electromagnetic shielding [20,21,22]. Emphasis has been placed on the creation of flexible and efficient materials able to protect against electromagnetic interference, especially polymer nanocomposites reinforced with carbon nanotubes [23,24,25]. These materials are light, corrosion-resistant, easily processable, and flexible.

Electromagnetic interference (EMI) is a type of environmental pollution caused by natural phenomena and electronic devices [26,27]. The broad development of electronic systems has brought out many concerns regarding electromagnetic pollution. Radio and microwave radiations are emitted by all electronic devices, particularly those operating in the radio wave and microwave frequency range [28,29,30]. A major concern is the interference of electromagnetic radiation with electronics by the interaction of electrons with the electric field of the radiation [31,32]. Electromagnetic interference is one of the most undesirable by-products of telecommunication devices and electronic systems, causing, in some cases, malfunction in electronic components [33,34]. In this context, the literature shows many advances in protective materials against electromagnetic radiation, i.e., shielding against electromagnetic interference (EMI) [35,36]. Nanocomposites are versatile materials with potential for electromagnetic shielding applications, especially in electronic devices. Some studies [37,38,39] have reported the high shielding efficiency of polyamide/conductive nanofiller systems against EMI. However, their preparation for EMI shielding is mainly done by directly mixing conductive fillers and polyamide. A single material can rarely satisfy all the necessary technical requirements of mechanical and electrical properties for special applications. Therefore, developing tough and highly conductive nanocomposites is of great scientific interest. It can result in new materials with properties tailored to multifunctional applications.

Flexible electronic materials are highly needed for various applications, such as robots, wearable electronics, and other devices. Likewise, flexible materials for electromagnetic interference (EMI) shielding with high mechanical and electrical performance are required to avoid the adverse effects of electromagnetic radiation produced by electronic devices [40,41]. In this context, Nylon 6/ABS polymer blends are highly regarded for their superior mechanical properties when compatibilized with a suitable reactive copolymer, and thus, are of prime importance in polymer technology. Nevertheless, Nylon 6/ABS blends are insulating in nature and need conductive nanofillers (carbon black, graphene, or carbon nanotubes) to improve their electrical properties and make high-performance nanocomposites for antistatic and magnetic shielding applications. Carbon nanotube-reinforced Nylon 6/ABS nanocomposites show a good correlation between electrical conductivity, rheology, and morphology [42]. However, adding a compatibilizing agent functionalized with maleic anhydride optimizes the carbon nanotube/Nylon 6/ABS properties. There is a lack of scientific literature on the potential of compatibilized Nylon 6/ABS nanocomposites for application in EMI shielding. Therefore, this represents a relevant investigation topic that can contribute to expanding the database regarding nanocomposites. An effective compatibilizing agent for Nylon 6/ABS blends is maleic anhydride-grafted acrylonitrile-butadiene-styrene (ABS-g-MA) since maleic anhydride is able to react with the amine end-groups of Nylon 6 and at the same time be miscible with ABS.

Nylon 6/ABS nanocomposites compatibilized with ABS-g-MA are industrially relevant. They can be applied in the automotive and electronics industry sectors. However, research on the effectiveness of Nylon 6/ABS/ABS-g-MA against electromagnetic interference has not been explored in the literature and constitutes a relevant investigation point. Therefore, the purpose of the present research is to develop Nylon 6/ABS nanocomposites compatibilized with ABS-g-MA and reinforced with carbon nanotubes for application against electromagnetic interference.

## 2. Results and Discussion

### 2.1. Rheological Properties

**Figure 1:**
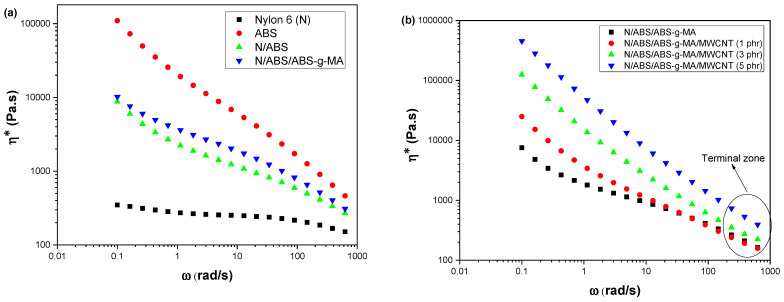
Complex viscosity of Nylon 6, ABS, blends, and nanocomposites as a function of increasing MWCNT content. (**a**) Nylon 6, ABS and blends; (**b**) Nylon 6/ABS/ABS-g-MA blend and nanocomposites with different MWCNT contents. N = Nylon 6.

Figure 1a illustrates the complex viscosity curves (η*) as a function of the angular frequency (ω) for Nylon 6, ABS, and their blends. The employed test conditions were: gap between plates of 1 mm, a temperature of 230 °C, and 2% deformation. It was observed that Nylon 6 has a typical Newtonian fluid behavior, with a constant plateau in practically the entire analyzed interval. In this case, the complex viscosity is frequency independent. On the other hand, ABS presented a higher complex viscosity that tended to continuously decrease at higher angular frequencies, suggesting a pseudoplastic characteristic. The blend viscosities were intermediate between the pure components (Nylon 6 and ABS) and superior to that of Nylon 6. The Nylon 6/ABS and Nylon 6/ABS/ABS-g-MA blends followed a pseudoplastic behavior. In addition, the Nylon 6/ABS/ABS-g-MA blend exhibited complex viscosity slightly higher than the N/ABS binary system, suggesting that the reactive compatibilizer (ABS-g-MA) increased phase interaction. Part of the ABS-g-MA molecular structure is miscible with ABS, and, at the same time, maleic anhydride can react with the terminal amine groups of Nylon 6. The miscibility of these groups can increase the synergistic effect between components, generating good mechanical properties. According to the literature [43,44], maleic anhydride groups react with the amine functional groups of Nylon 6 to form imide groups. 

Figure 1b presents the complex viscosity curves (η*) as a function of angular frequency (ω) for the Nylon 6/ABS/ABS-g-MA blend and nanocomposites. Because the Nylon 6/ABS/ABS-g-MA/MWCNT nanocomposites were hard to melt, test conditions were adjusted as follows: 2 mm gap, 240 °C, and 2% deformation. For comparison, similar conditions were employed to investigate the rheological behavior of the Nylon 6/ABS/ABS-g-MA blend. At low frequency (<1 rad/s), the viscosity of the nanocomposites changed to much higher values, which was also observed by other authors [45,46,47]. MWCNT addition into the Nylon 6/ABS/ABS-g-MA system drastically increased the complex viscosity and intensified the pseudoplastic behavior. The complex viscosity of the nanocomposites continuously increased by incrementing MWCNT content, which hindered melt flow during processing. Such behavior can be attributed to the increased friction between the polymer matrix and the conductive charge, which restricts molecular movement in the polymer matrix [48]. Accordingly, the material has less fluidity, becoming more rigid and behaving like an elastic solid [49,50]. According to reports in the literature [51,52], the preferential location of carbon nanotubes can affect the viscosity in multicomponent polymeric materials. Nanofillers incorporated in the polymer matrix led to higher viscosities than when present in the dispersed phase. Therefore, the preparation of Nylon 6/ABS/ABS-g-MA/MWCNT mixtures suggests a preferred location of MWCNT in Nylon 6, resulting in high complex viscosity values. 

At high frequencies, the Nylon 6/ABS/ABS-g-MA/MWCNT nanocomposites approximate the value of complex viscosity compared to the Nylon 6/ABS/ABS-g-MA system. Stanciu et al. [53] reported that, at higher frequencies, the chains unfold and align in the flow direction. Furthermore, with the collapse in the molecular structure of the mixture, the carbon nanotubes are oriented in the flow direction (become aligned), thereby producing a decrease in viscosity. Figure 1b shows that the nanocomposite with 5 phr MWCNT presented the highest complex viscosity in the terminal zone, revealing a better dispersion of the conductive additive in the Nylon 6 matrix. The literature [54] reported a similar behavior and attributed the viscosity increment to a high dispersion state of the nanofiller. This helps to improve the matrix-nanofiller interfacial contact area and, consequently, produces a higher viscosity.

**Figure 2:**
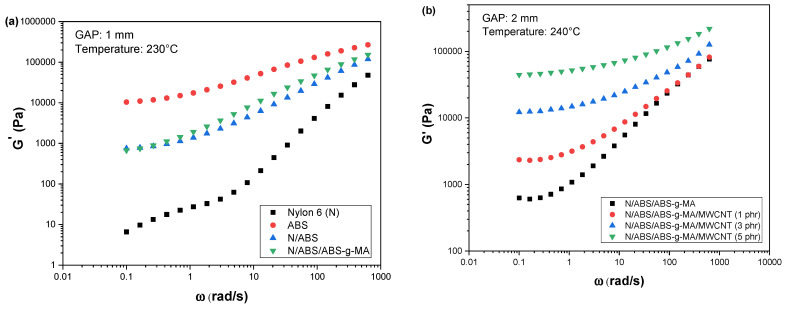
Storage modulus curves of Nylon 6, ABS, blends, and nanocomposites. (**a**) Nylon 6, ABS and blends; (**b**) Nylon 6/ABS/ABS-g-MA blend and nanocomposites with different MWCNT contents; N = Nylon 6.

Figure 2a,b shows the storage modulus (G’) curves as a function of the oscillation frequency for Nylon 6, ABS, and their blends and nanocomposites. Nylon 6 exhibited a viscous liquid behavior, while ABS showed a predominant characteristic of a pseudo-solid. The Nylon 6/ABS and Nylon 6/ABS/ABS-g-MA blends presented a slope tending to zero at low frequencies, which is a typical behavior of pseudo-solid materials.

Figure 2b shows a very significant increase in the G’ values of the Nylon 6/ABS/ABS-g-MA/MWCNT nanocomposites compared with the Nylon 6/ABS/ABS-g-MA blend. This behavior indicates an increment in the viscoelastic characteristics of the nanocomposites caused by MWCNT. The Nylon 6/ABS/ABS-g-MA/MWCNT systems presented increased G’ values with increasing nanofiller content, mainly in the low-frequency region, which is typical of polymers loaded with conductive particles [55,56]. The nanocomposites containing 3 and 5 phr MWCNT presented higher G’ values than the sample with 1 phr MWCNT, following the same behavior observed for η*. Furthermore, the slope decrease of the G’ curves at low frequencies, especially for 3 and 5 phr, indicates the formation of a more effective percolated network structure for these compositions [57]. The result was better electrical conductivity and more effective electromagnetic shielding, as will be seen later. Apparently, the Nylon 6/ABS/ABS-g-MA/MWCNT nanocomposites from 1 phr MWCNT onwards showed a slope decrease tendency. This indicates an established percolated filler network in the nanocomposites. In the high-frequency regime, except for the sample with 5 phr MWCNT, the nanocomposites and the Nylon 6/ABS/ABS-g-MA blend exhibited similar storage modulus. The high frequency corresponds to motion on a small-time scale, indicating that molecular motions were not severely affected by the addition of MWCNT [58]. 

### 2.2. Electrical Properties

**Figure 3:**
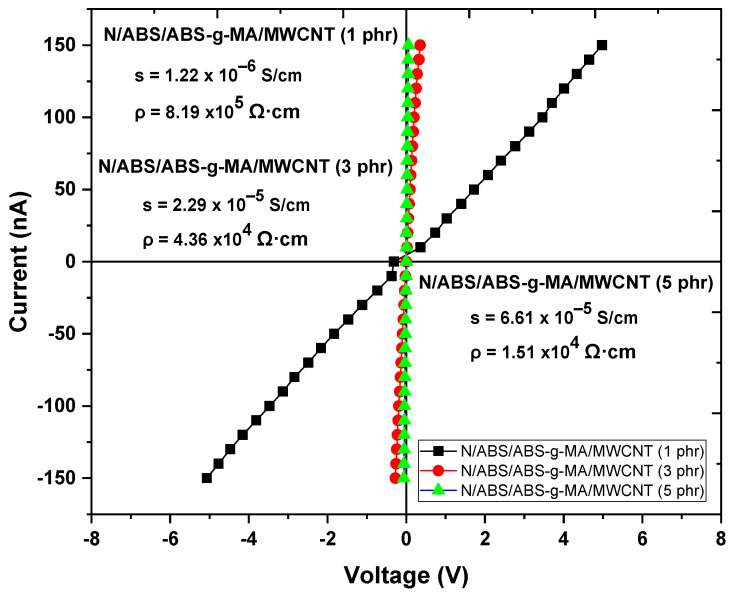
Electrical behavior of the nanocomposites in the –7 V to 7 V range. N = Nylon 6.

Figure 3 shows the electrical behavior of the Nylon 6/ABS/ABS-g-MA/MWCNT nanocomposites obtained through current-voltage (I-V) studies. Conductivity tests were not performed on Nylon 6 and ABS because they are typically isolating materials. The nanocomposites show a linear response in the evaluated –7 V to 7 V range, indicating an ohmic behavior. The rise of the I-V curve slope suggests improvement in electrical conductivity with the increase in MWCNT concentration. In other words, there was a drop in the electrical resistance of the nanocomposites. Such behavior demonstrates that small concentrations of MWCNT in insulating materials may promote a high electrical conductivity. The nanocomposites loaded with 3–5 phr MWCNT exhibited a more severe ohmic response. They present symmetric curves and a bias voltage lower than 0.5 V. These nanocomposites showed a more efficient electrical percolation path (discussed in the rheology test), which contributed to a faster electrical response and lower voltage. In comparative terms, the Nylon 6/ABS/ABS-g-MA/MWCNT nanocomposite (5 phr) showed a slightly higher electrical response compared with the system containing 3 phr.

Figure 3 shows the electrical conductivity (σ) and resistivity (ρ) of the Nylon 6/ABS/ABS-g-MA/MWCNT nanocomposites. As observed, the electrical conductivity of the Nylon 6/ABS/ABS-g-MA/MWCNT nanocomposites increased continuously with increasing MWCNT concentration. They exhibit a typical behavior associated with the electrical percolation phenomenon [59]. Nanocomposites with 1 phr MWCNT showed electrical conductivity and resistivity of 1.22 × 10^−6^ S/cm and 8.19 Ω·cm, respectively. The 3 phr and 5 phr MWCNT concentrations promoted a more significant increase in electrical conductivity, leading to values of 2.29 × 10^−5^ S/cm and 6.61 × 10^−5^ S/cm, respectively. These results show that a higher MWCNT content promoted higher conductivities due to a percolated network of nanotubes. This is supported by the rheological studies that showed a noticeable viscosity increment at low frequencies.

From a practical perspective, the electrical properties of the materials suggest a high potential for antistatic packaging application. Products with antistatic characteristics arouse interest in technological applications, especially in the packaging of electronic circuits and the dispersion of static charge to avoid damage to more sensitive electronic equipment, as well as possible sparks arising from a potential difference when in contact with flammable liquids [60,61]. According to Ribeiro et al. [62], materials having potential for antistatic packaging should present electrical conductivity higher than 10^−8^ S/cm. In this case, the nanocomposites developed in our work can be applied in the manufacture of products for static charge dissipation. The 1 phr MWCNT concentration proved suitable for antistatic applications. It improved the main properties without a high nanofiller content, enabling production at a low cost.

Electrical resistivity indicates how much a material opposes the passage of an electric current. The higher the electrical resistivity of a material, the more difficult it is for an electric current to pass through. As shown in Figure 3, the Nylon 6/ABS/ABS-g-MA/MWCNT nanocomposites with 3 and 5 phr MWCNT have the lowest electrical resistivity. Furthermore, the electrical resistivity of the nanocomposites with 1, 3, and 5 phr are within the range for antistatic application (10^4^–10^11^ Ω·cm) [63].

### 2.3. Electromagnetic Shielding

**Figure 4:**
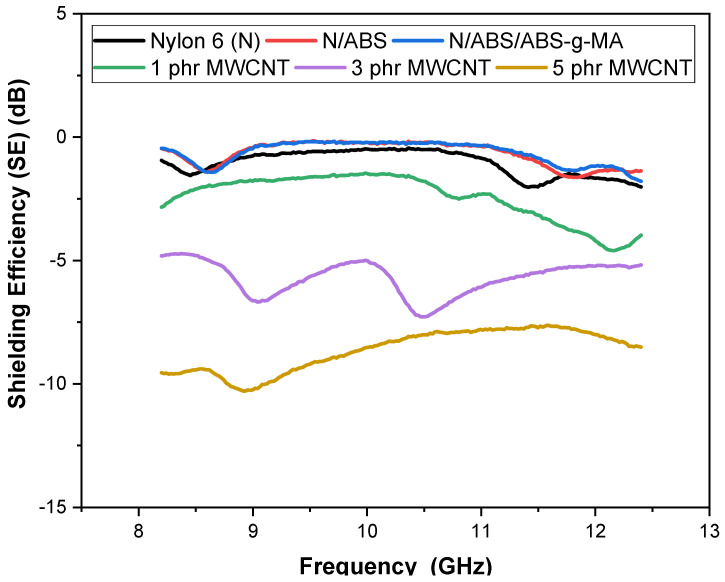
Shielding efficiency as a function of frequency for Nylon 6, blends, and nanocomposites. N = Nylon 6.

Figure 4 presents the electromagnetic radiation shielding efficiency (EMI-SE) versus frequency for Nylon 6, blends, and nanocomposites. Nylon 6, Nylon 6/ABS, and Nylon 6/ABS/ABS-g-MA blends showed practically no attenuation of the electromagnetic radiation given their insulating nature. The addition of 1 phr MWCNT led to a subtle increase in the attenuation degree compared with the insulating materials. Because carbon nanotubes boost the electrical conductivity and, consequently, the interaction level with electromagnetic radiation, they have been used as materials for the manufacture of nanocomposites with a high EMI shielding effect [64]. In the 8.5 GHz to 10.5 GHz range, the Nylon 6/ABS/ABS-g-MA/MWCNT nanocomposite (1 phr) showed stable attenuation around –1.6 dB. Above 10.5 GHz, the Nylon 6/ABS/ABS-g-MA/MWCNT showed increased electromagnetic radiation attenuation power, reaching a peak of –4.5 dB for 12 GHz. Such behavior indicates that, depending on the analyzed frequency, the attenuation effect can vary for Nylon 6/ABS/ABS-g-MA/MWCNT nanocomposites.

The total attenuation of electromagnetic radiation improved as the MWCNT incremented in the Nylon 6/ABS/ABS-g-MA/MWCNT systems (3 and 5 phr). This behavior may be ascribed to the higher amount of dispersed nanocarbons that led to higher conductivities and better interaction with electromagnetic radiation. Similar behavior was reported in the literature [65,66], indicating that increasing the amount of conductive additive in a polymer matrix improves the electromagnetic shielding efficiency. A higher amount of conductive additive favors the electrical conductivity and, concurrently, additive interaction with the electromagnetic radiation, tuning the shielding efficiency. Figure 4 shows that, in the 8 to 10 GHz range, an intense oscillation dominated the electromagnetic shielding behavior of the nanocomposite with 3 phr MWCNT. This frequency range is critical given the instability of electromagnetic shielding in this region. However, in the 10.5 to 12 GHz range, there was higher stability near –5.5 dB. The best magnetic shielding efficiency was obtained for the composition containing 5 MWCNT, possibly due to the higher electrical conductivity, as discussed in Figure 3. The nanocomposite with 5 MWCNT presented the highest electrical conductivity (6.61 × 10^−5^ S·cm^−1^) and, hence, a more efficient and stable electromagnetic shielding. It showed a peak around –10.5 dB at 9 GHz and an almost steady value near –8.2 dB in the 11 to 12 GHz frequency range.

**Figure 5:**
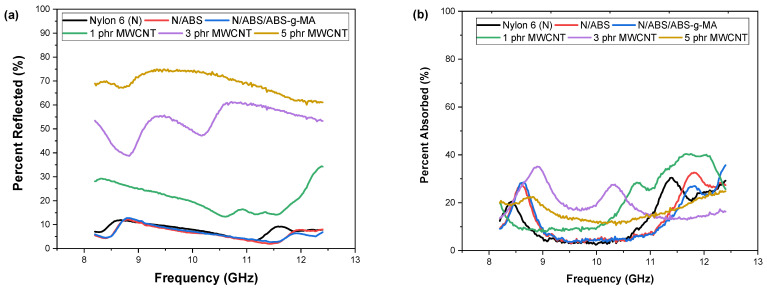
Contribution of reflection and absorption to electromagnetic wave attenuation for Nylon 6, blends, and nanocomposites. (**a**) Percentage by reflection for Nylon 6, ABS, blends and nanocomposites. (**b**) Percentage by absorption for Nylon 6, ABS, blends and nanocomposites. N = Nylon 6.

To understand the predominant shielding mechanism, Figure 5a,b presents the percentage contribution of absorption and reflection to the attenuation of electromagnetic radiation. The absorption and reflection mechanisms were comparable for nanocomposites with up to 1 phr MWCNT. Although the lower MWCNT amount provides an established conductive network (Figure 3), the structure probably had spaces that could facilitate the penetration of electromagnetic radiation. At the same time, nanotubes on the surface could interact with the radiation. As a consequence, there was a balanced mechanism between absorption and reflection. Meanwhile, the shielding mechanism showed a different behavior for the Nylon 6/ABS/ABS-g-MA/MWCNT nanocomposites containing 3 phr and 5 phr MWCNT, especially for 5 phr MWCNT. The contribution of the reflection mechanism was predominant for samples with 3 phr and 5 phr MWCNT (percentages ranged from 40 to 75%). Therefore, the main attenuation phenomenon for nanocomposites with 3 phr and 5 phr involves reflection, which is possibly due to a dense conductive network increasing charge carrier concentration. The charge carriers interact directly with the incident wave, contributing to the reflection mechanism. The maximum contribution of the reflection mechanism was attained for nanocomposites with 5 phr MWCNT, probably due to the higher electrical conductivity that directly contributed to improving the electromagnetic shielding. Furthermore, the Nylon 6/ABS/ABS-MA/MWCNT system (5 phr) exhibited a low absorption level associated with the low transmitted power and higher reflectivity.

**Figure 6:**
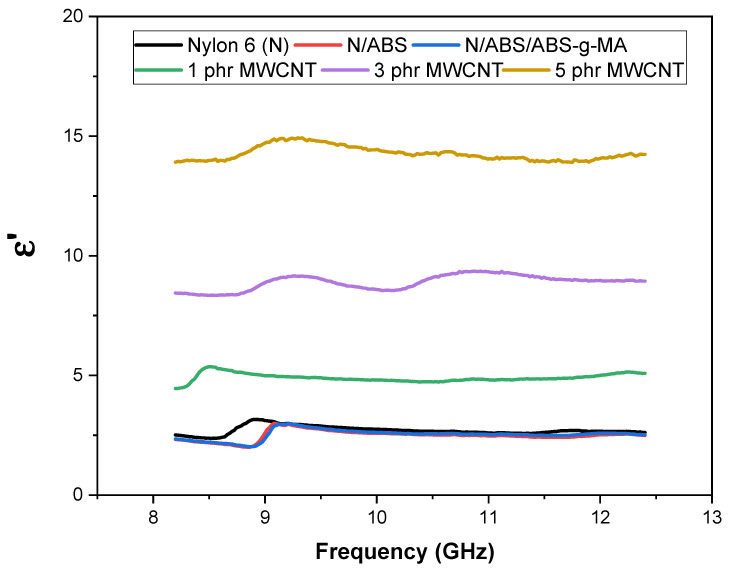
Behavior of the dielectric permittivity as a function of frequency for Nylon 6, blends, and nanocomposites. N = Nylon 6.

**Figure 7:**
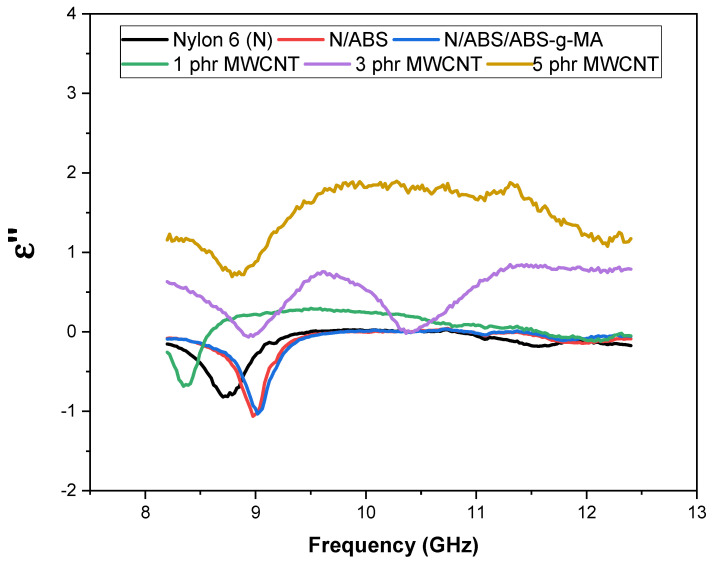
Behavior of dielectric permittivity loss (ε”) as a function of frequency of Nylon 6, blends and nanocomposites. N = Nylon 6.

The absorption mechanism of the electromagnetic shielding efficiency can be better understood through the permittivity (ε) vs. frequency curves. Dielectric permittivity (ε’) represents the contribution of the polarization mechanisms of electric charges. In general, an increase in ε´ can be attributed mainly to the polarization effect [67]. The absorption shielding efficiency is related to high values of ε´. Figure 6 shows the behavior of the dielectric constant (ε′) for Nylon 6, blends, and nanocomposites in the 8 to 12 GHz frequency range. Nylon 6 and blends presented the lowest dielectric permittivity (ε′) values due to their insulative nature. The dielectric constant (ε′) increased with MWCNT increment in the Nylon 6/ABS/ABS-g-MA system. This was due to the presence of conductive nanofillers (MWCNT) that improved polarization. As expected, the permittivity (ε′) of the Nylon 6/ABS/ABS-g-MA with 5 phr MWCNT was the most expressive due to its high electrical conductivity, which caused strong polarization and intense dissipation of electrostatic charges. In addition, the dielectric constant (ε′) in the analyzed frequency range was practically stable, indicating that the dominant polarization mechanism is the dipole [68]. Figure 7 shows the behavior of the dielectric loss (ε″) for Nylon 6, blends, and nanocomposites. This parameter represents the contribution of the conduction mechanism caused by the polarization of the material. Therefore, the values of ε″ are related to the energy dissipation of the incident electromagnetic waves. It was observed that the increment in MWCNT content provided an increase in ε″, especially for the 3 and 5 phr concentrations. The nanocomposite containing 5 phr of MWCNTs showed the highest ε″ performance, with a practically constant value and with small fluctuations along the studied frequency band, except around 9 GHz. The improved ε″ can be attributed to the high conductivity of the nanocomposites. Consequently, the incorporation of MWCNTs in a larger amount in the Nylon 6/ABS/ABS-g-MA system was more effective at attenuating the electromagnetic radiation. Because of this, the Nylon 6/ABS/ABS-g-MA/MWCNT (5 phr) nanocomposite showed the highest efficiency dissipating the energy of the incident waves, justifying the higher electromagnetic shielding. 

### 2.4. Scanning Electron Microscopy (SEM)

**Figure 8:**
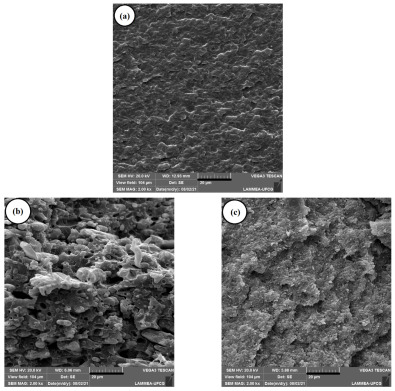

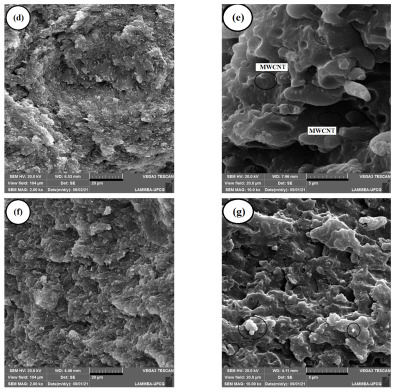

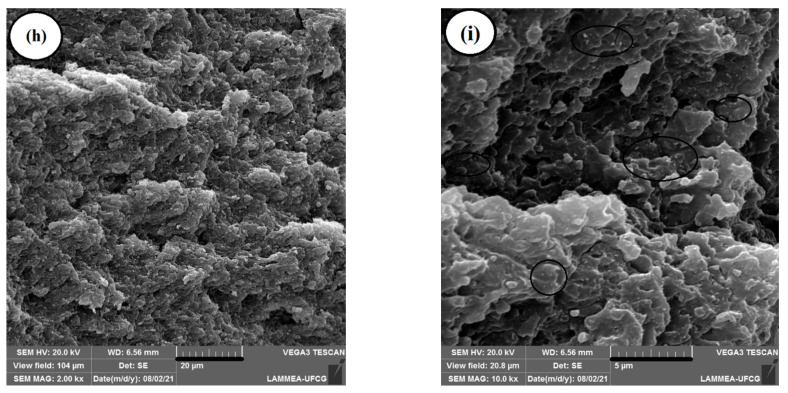
SEM micrographs of the impact test fracture surface: (**a**) Nylon 6 (2000×); (**b**) Nylon 6/ABS (2000×); (**c**) Nylon 6/ABS/ABS-g-MA (2000×); (**d**,**e**) Nylon 6/ABS/ABS-g-MA/MWCNT (1 phr) (2000× and 10,000×); (**f**,**g**) Nylon 6/ABS/ABS-g-MA/MWCNT (3 phr) (2000× and 10,000×); (**h**,**i**) Nylon 6/ABS/ABS-g-MA/MWCNT (5 phr) (2000× and 10,000×).

Figure 8a–c shows SEM micrographs of Nylon 6, the Nylon 6/ABS, and Nylon 6/ABS/ABS-g-MA blends, respectively. Figure 8a shows the fracture surface of Nylon 6. Nylon 6 presents a ductile fracture aspect with plastic deformation. The Nylon 6/ABS blend (60/40%) (Figure 8b) exhibits dispersed ABS particles of various sizes and some holes representing pulled-out ABS particles extracted from the Nylon 6 surface during the impact test. Also, the degree of interfacial adhesion was low between Nylon 6 and ABS, indicating a low interfacial strength caused by the structural difference. There is morphological evidence of immiscibility between Nylon 6 and ABS, characterized by the poor adhesion between phases. The immiscibility reduced the mechanical properties of impact strength and elongation at break (presented later).

The compatibilization of Nylon 6/ABS with 10% ABS-g-MA promoted the formation of a more stable morphology (see Figure 8c). Clearly, the ABS particles dispersed in the Nylon 6 matrix suffered a significant size reduction, improving the interfacial adhesion between phases. The literature [69] reported that an efficient polymer blend compatibilizer allows diffusion to the phase interface, reducing the interfacial energy and preventing the particles from coalescing. Such behavior is fundamental to improving the toughening mechanism and, consequently, the flexibility and the degree of energy dissipation. Reactive compatibilization was possible because ABS-g-MA is miscible with ABS and the maleic anhydride group reacts with the amine end groups of Nylon 6. Compatibilization of the Nylon 6/ABS/ABS-g-MA system was effective, generating a refined morphology with well-adhered particles and producing a synergistic effect in the mechanical properties.

As shown in Figure 8d–i, the morphology of the Nylon 6/ABS/ABS-g-MA/MWCNT nanocomposites indicated the maintenance of a ductile behavior with the fracture surface, exhibiting a characteristic plastic deformation. The dispersion and distribution of nanofillers improved with incremental MWCNT content. These results further endorse the oscillatory rheology and electrical conductivity analyses. The exception was the Nylon 6/ABS/ABS-g-MA/MWCNT system (3 phr), which apparently showed a low cluster level (see Figure 8g). Furthermore, the Nylon 6/ABS/ABS-g-MA/MWCNT nanocomposites kept their morphological characteristics stable, i.e., fine ABS particles remained with practically no observable holes. Bose et al. [42] reported similar behavior. They noticed the formation of a refined morphology of Nylon 6/ABS blends with carbon nanotubes, especially in higher concentrations. It was suggested that the ABS phase refinement occurred due to the preferential location of carbon nanotubes in the Nylon 6 phase. Thus, the incorporation of carbon nanotubes can contribute to breaking the ABS phase droplets to produce fine particles. Apparently, the Nylon 6/ABS/ABS-g-MA/MWCNT nanocomposites showed refined morphologies due to the preferential migration of MWCNT into the Nylon 6 phase, contributing to the maintenance of good mechanical properties. 

**Figure 9:**
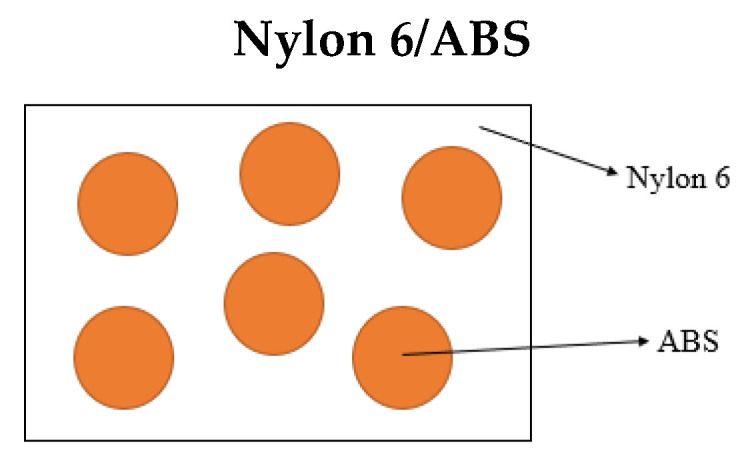

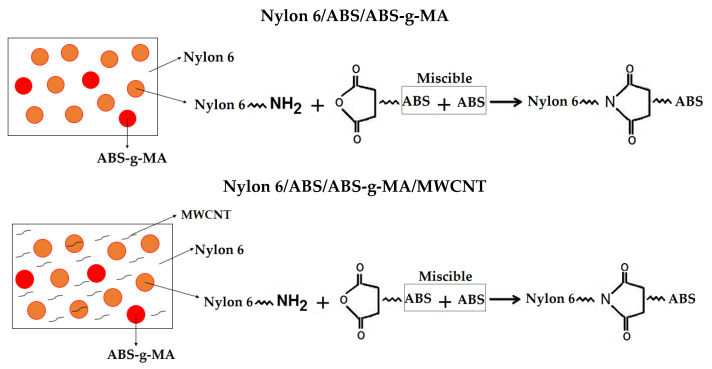
Schematic representation of the blends and nanocomposites.

Figure 9 illustrates the compatibilization mechanism of the blends and nanocomposites. The Nylon 6/ABS mixture has no molecular interaction given their different structures. The high concentration of ABS (40%) in the Nylon 6 matrix promoted the formation of a coarse morphology with large particles, which can be attributed to the ABS phase coalescence phenomenon. This behavior has been reported by Majumdar et al. [70] for Nylon 6/ABS mixtures, suggesting that ABS droplets can coalesce at high temperatures when Nylon 6 has a very low viscosity. For the Nylon 6/ABS/ABS-g-MA system, the average diameter of the ABS particles was smaller due to the migration of ABS-g-MA to the Nylon 6/ABS interface. One of the effects of the compatibilizer is to minimize the interfacial tension between the dispersed and matrix phases, facilitating mutual phase dispersion. Furthermore, the compatibilizer can improve interaction and adhesion between the Nylon 6 and ABS phase boundaries, enhancing phase stability and preventing coalescence [71,72]. However, during Nylon 6/ABS compatibilization, part of the ABS-g-MA may remain dispersed as a third phase, participating in the toughening mechanism. The Nylon 6/ABS/ABS-g-MA/MWCNT nanocomposites have similar morphology to the Nylon 6/ABS/ABS-g-MA base mixture, except for the dispersed carbon nanotubes. The presence of a rigid nanofiller dispersed in the Nylon 6/ABS/ABS-g-MA system deteriorates the toughening mechanism, reducing its flexibility. On the other hand, it favors the electrical conductivity and electromagnetic shielding mechanism. The stable nanocomposite morphology presented in Figure 8d–i led to a balance of properties and allowed the production of flexible and conductive materials for application in the electrical industry.

### 2.5. Mechanical Properties

**Figure 10:**
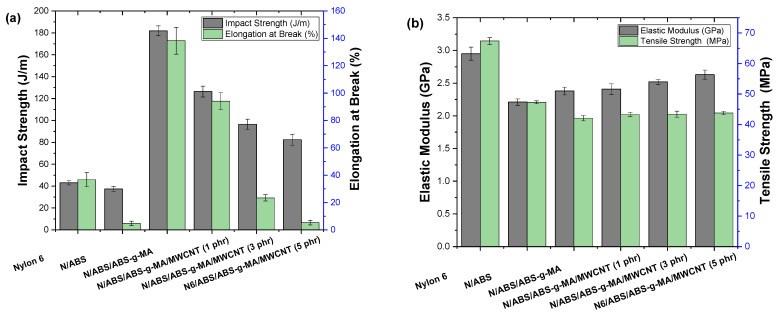
Mechanical properties of Nylon 6, blends, and nanocomposites: (**a**) impact strength vs. elongation at break; (**b**) elastic modulus vs. tensile strength. N = Nylon 6.

Figure 10 shows the mechanical properties of Nylon 6, blends, and nanocomposites as a function of MWCNT concentration. The elastic modulus, impact strength, tensile strength, and elongation at break are summarized in Table 1. Nylon 6 presented a low impact strength (about 43 J/m) for an engineering polymer. The presence of 40% ABS was not able to toughen the Nylon 6 specimens, reducing the impact strength to 37.4 J/m. SEM analysis of the Nylon 6/ABS mixture showed poor interfacial adhesion, evidencing an inefficient energy dissipation mechanism. The ABS-g-MA compatibilizer substantially contributed to the toughening mechanism in light of the high impact strength presented by the Nylon 6/ABS/ABS-g-MA mixture. This system exhibited an impact strength of about 181.9 J/m, typical of toughened materials at room temperature. In quantitative terms, the Nylon 6/ABS/ABS-g-MA blend incremented the impact strength by around 323% and 387% compared with Nylon 6 and the non-compatibilized system, respectively. Maleic anhydride, present in the ABS-g-MA used to modify the Nylon 6/ABS system, reacted with the amine end groups of Nylon 6, remaining miscible with the ABS phase and ensuring reactive compatibilization. As a result, the energy dissipation mechanism improved, leading to the high impact strength response. MWCNT increment in the nanocomposites caused a continuous impact strength reduction compared with the Nylon 6/ABS/ABS-g-MA base mixture. The literature [73] indicated that carbon nanotubes act as stress concentrators and restrict the molecular mobility of nanocomposites, consequently reducing the impact strength. However, the Nylon 6/ABS/ABS-g-MA/MWCNT nanocomposites showed a significantly higher impact strength than Nylon 6. The nanocomposites containing 5 phr MWCNT showed an impact strength of 82.4 J/m, an improvement of 91% compared with Nylon 6. Therefore, even the higher MWCNT concentration formed toughened nanocomposites at room temperature. As proposed (Figure 9), the mobility of the Nylon 6/ABS/ABS-g-MA system was counterbalanced by the MWCNT dispersion, producing materials with good impact strength properties.

Table 1 shows that Nylon 6 presented the highest rigidity, suggesting a higher resistance to elastic deformation. Nylon 6/ABS and Nylon 6/ABS/ABS-g-MA blends had a lower elastic modulus than Nylon 6. This is due to the higher flexibility provided by the ABS phase. The Nylon 6/ABS/ABS-g-MA system had a slightly increased elastic modulus (2.38 GPa) compared with the non-compatibilized mixture. The addition of MWCNT in the Nylon 6/ABS blend led to an incremental increase in elastic modulus, observable for all nanocomposites. The highest elastic modulus was obtained with 5 phr MWCNT, an increment of 10.5% compared with the Nylon 6/ABS/ABS-g-MA base mixture. However, the values found for Nylon 6/ABS/ABS-g-MA/MWCNT systems, regardless of MWCNT content, are lower than those of Nylon 6.

Table 1 shows that Nylon 6 presented higher tensile strength and required a high load to deform (~67.4 MPa). The tensile strength of the Nylon 6/ABS blend decreased to 47.3 MPa compared with Nylon 6. The compatibilization of Nylon 6/ABS by ABS-g-MA promoted a decrease in the tensile strength to 42.1 MPa. The drop in tensile strength was caused by the higher ductility that led to deformation at lower stresses. A slight increase in the tensile strength of the Nylon 6/ABS/ABS-MA/MWCNT nanocomposites was observed compared with the Nylon 6/ABS/ABS-g-MA base system. The tensile strength of the nanocomposites reached a maximum value with 5 phr of MWCNT (43.8 MPa). Apparently, carbon nanotubes (MWCNT) acted subtly as a reinforcing agent, receiving part of the mechanical stress. At the same time, increasing the MWCNT concentration did not seem to significantly influence the tensile strength of Nylon 6/ABS/ABS-MA/MWCNT nanocomposites, as the values are within the experimental error margin.

The elongation at break of Nylon 6 was about 36.7%, while, with the addition of 40% ABS, it decreased to 4.8%. The significant reduction in the elongation at break of the Nylon 6/ABS blend reveals the incompatibility of this system, corroborating with the SEM analysis. The ABS-g-MA compatibilizer had a considerable influence on the value of elongation. A significantly high elongation at break was reached with the Nylon 6/ABS/ABS-g-MA blend (a strain of 138.3%), corresponding to an increase of 276.8% and 2781% compared with Nylon 6 and the Nylon 6/ABS mixture, respectively. This is evidence that ABS-g-MA improved the deformation mechanism and increased the ductility of Nylon 6/ABS. The complex viscosity curves suggested that the ABS-g-MA reacted with the amine groups of Nylon 6 while remaining miscible with the ABS phase, guaranteeing blend compatibilization. As a result, there was a synergistic effect on the Nylon 6/ABS/ABS-g-MA system, improving the deformation and the degree of flexibility. MWCNT addition to the Nylon 6/ABS/ABS-g-MA mixture drastically affected the elongation at break. As the MWCNT content increased, the elongation at break decreased continuously, especially for 5 phr MWCNT. The preparation of Nylon 6/ABS/ABS-g-MA/MWCNT nanocomposites (up to 1 phr MWCNT) still kept a high elongation at break that was even superior to Nylon 6. However, with 3 and 5 phr MWCNT, the elongation at break was severely reduced, suggesting loss of ductility. Therefore, the 1 phr MWCNT concentration is critical for the nanocomposites, considering that, above it, the elongation at break was inferior to that of Nylon 6. Similar behavior was observed in the literature [74], wherein carbon nanotube concentrations above 1% inhibit the deformation process of the ductile matrix due to the large difference between the elastic modulus of the nanofiller and the thermoplastic matrix.

**Figure 11:**
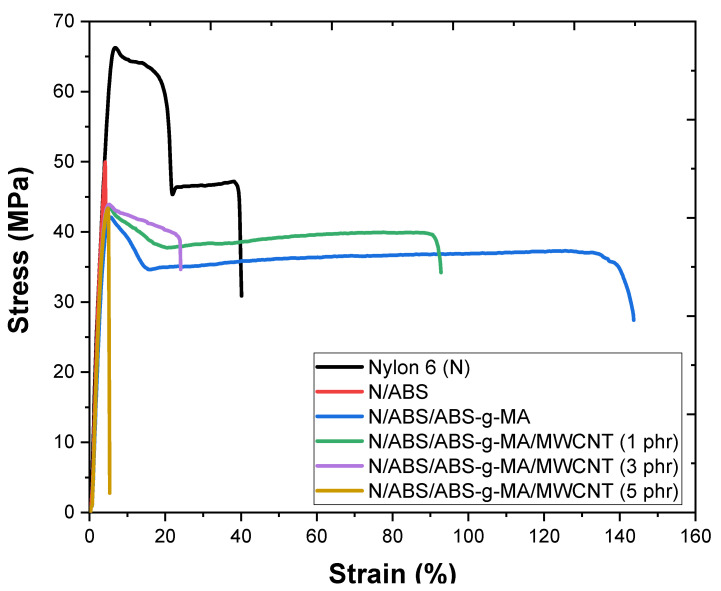
Stress-strain curves of Nylon 6, blends, and nanocomposites as a function of MWCNT concentration. N = Nylon 6.

Figure 11 shows the stress-strain curves of Nylon 6, blends, and nanocomposites as a function of MWCNT concentration. Nylon 6 showed a ductile polymer behavior, with plastic yielding and high elongation. The Nylon 6/ABS blend is distinctively a fragile mixture, presenting no flow and practically no elongation at break. The Nylon 6/ABS compatibilization with ABS-g-MA considerably improved the elongation at break, which was evidenced by the large area under the stress-strain curve (typical of toughened materials). On the other hand, the Nylon 6/ABS/ABS-g-MA/MWCNT nanocomposites presented a continuous reduction in strain level. The ductile and tenacious behavior was preserved with the addition of a low nanofiller concentration (1 phr). However, lower deformation at fracture was observed compared with the Nylon 6/ABS/ABS-g-MA blend. The higher MWCNT content (3 and 5 phr) led to a severe reduction in strain. Prashantha et al. [75] reported that strain reduction in carbon nanotube-reinforced nanocomposites is associated with the formation of agglomerates, generating stress concentration, and reducing the tensile strain capacity. Therefore, a high concentration of nanotubes supports polymer deformation (under tensile) at preferential locations, concentrating stress and inhibiting an efficient deformation mechanism.

### 2.6. Thermogravimetry (TG)

**Figure 12:**
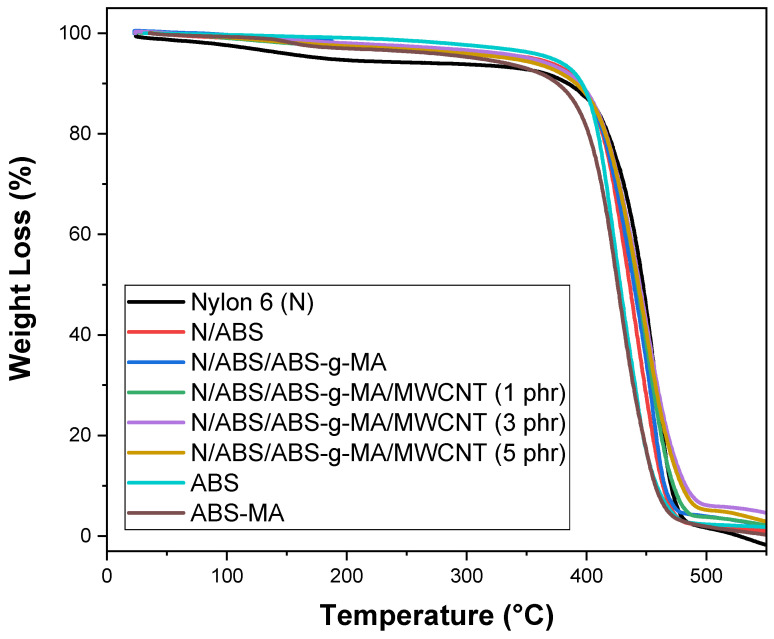
TG curves of Nylon 6, Nylon 6/ABS, Nylon 6/ABS/ABS-g-MA, and nanocomposites as a function of carbon nanotube content. N = Nylon 6.

The weight loss curves of the pure polymers, Nylon 6/ABS Nylon 6/ABS, Nylon 6/ABS/ABS-g-MA, and the nanocomposites are shown in Figure 12. Nylon 6 showed a decline in the TG curve between 30 and 150 °C, possibly due to the presence of humidity. In this temperature range, ABS and ABS-g-MA showed better thermal stability than Nylon 6. The Nylon 6/ABS and Nylon 6/ABS/ABS-g-MA blends and the nanocomposites preserved the thermal stability between 30 to 150 °C, given that there was no material loss. ABS addition to Nylon 6 created a barrier effect, reducing moisture absorption and directly increasing thermal stability in the range of 30–150 °C. At 550 °C (final temperature), the nanocomposites showed residues of carbon nanotubes, which typically do not decompose completely at this temperature.

Figure 12 shows that Nylon 6, the blends, and nanocomposites have only one decomposition stage, representing practically 100% of the entire mass loss. This weight loss is ascribed to the depolymerization process, i.e., the degradation of the polymeric chains. Nylon 6 shows a curve shift to higher temperatures beginning at 420 °C, indicating superior thermal stability at high temperatures. Blends and nanocomposites exhibit thermal decomposition behaviors intermediate to the pure components at temperatures above 400 °C.

The Nylon 6/ABS/ABS-g-MA blend showed a subtle shift in the TG curve from 420 °C onwards compared with the Nylon 6/ABS blend. Such behavior was probably due to the weak interactions between phases in the Nylon 6/ABS mixture, as seen in the mechanical results and the morphological analysis. On the other hand, the Nylon 6/ABS/ABS-g-MA blend suggests a synergistic interaction between the components of the mixture, generating a stabilizing effect. The thermogravimetric curves of the Nylon 6/ABS/ABS-g-MA blend reinforced with carbon nanotubes slightly shifted to higher temperatures, indicating an increase in thermal stability. However, the addition of carbon nanotubes to the Nylon 6/ABS/ABS-g-MA blend had little influence on the thermal decomposition behavior compared with Nylon 6.

Table 2 shows the temperatures for 10% (T_0.1_) and 50% (T_0.5_) mass loss obtained to evaluate the thermal stability of Nylon 6, blends, and nanocomposites. The temperature for 10% mass loss was higher for Nylon 6/ABS and Nylon 6/ABS/ABS-g-MA blends than for Nylon 6 due to the additive effect of more ABS. Regarding the Nylon 6/ABS/ABS-g-MA/MWCNT nanocomposites, T_0.1_ shifted to a higher temperature compared with Nylon 6 and the Nylon 6/ABS/ABS-g-MA blend. The dispersed carbon nanotubes help to delay the onset of thermal degradation. Chiu and Kao [76] pointed out that multi-walled carbon nanotubes increase the thermal stability of polyamide 46 by forming protective layers of dispersed nanotubes.

## 3. Materials and Methods

### 3.1. Materials

Nylon 6 (N) pellets supplied by ThaThi polymers (market code B300^®^, with density and flow rate of 1.13 g/cm^3^ and 2.9 g/10 min, respectively) were used as the polymer matrix (ThaThi polymers, São Paulo, Brazil).

Acrylonitrile-butadiene-styrene (ABS) terpolymer was supplied by Innova, commercial code AE8000^®^, density of 1.04 g/cm^3^, and flow rate of 5 g/10 min (Formosa Chemicals Industries, Taipei, Taiwan).

Maleic anhydride-grafted acrylonitrile-butadiene-styrene (ABS-g-MA), with a 3.1% maleic anhydride grafting degree (Formosa Chemicals Industries, Taipei, Taiwan), was used as compatibilizer. The detailed characterization of the ABS grafting process can be consulted in the literature [77].

Multi-walled carbon nanotubes (MWCNT) were prepared by chemical vapor deposition (CVD) in the form of black powder and supplied by Advanced 2D Materials. Specifications are: inner diameter: 3–5 nm; outside diameter: 8–15 nm; length: 3–12 μm; specific surface area: >233 m^2^/g; density: 0.15 g/cm^3^; and electrical conductivity of 100 s/cm (Advanced 2D Materials, Shanghai, China).

### 3.2. Methods

#### 3.2.1. Processing of Materials

Before blend preparation, Nylon 6 was dried in a vacuum oven at 80 °C for 24 h. ABS, ABS-g-MA, and the carbon nanotubes were dried in the same vacuum oven for 24 h at 60 °C. Table 3 presents the formulations of the blends and nanocomposites. The ABS content of 30 wt% was chosen to obtain toughened Nylon 6 and still maintain good stiffness. The literature has reported ABS percentages in the order of 30–50 wt% [19,42,70]. On the other hand, ABS-g-MA content was set at 10 wt%, considering that compatibilizer concentration exceeding this value incurs a higher cost.

The nanocomposites and blends were processed under an air atmosphere at 230 °C using a Thermo Scientific Haake Rheomix 3000 internal mixer set with roller-type rotors (Thermo Fisher Scientific, Waltham, MA, USA). The rotation speed employed during processing was 60 rpm. Nylon 6 was processed under the same conditions as the nanocomposites and blends for comparative purposes. The total mass (M_t_) added to the mixer was determined using Equation (1):

M_t_ = 0.7 × *ρ* × V_n_ (1)

where: *ρ* = material density; 0.7 = volume of polymer mass in the mixing chamber (70%); V_n_ = total volume of mixing chamber (310 cm^3^). After melt processing, all materials were ground using a knife mill.

**Figure 13:**
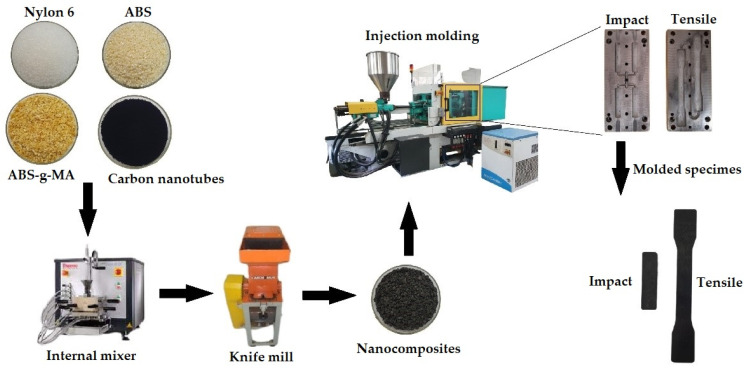
Representation of nanocomposite preparation.

The nanocomposites and blends were injection molded in an Arburg injection molding machine, Allrounder Model 207C Golden Edition (Arburg, Radevormwald, Germany), to obtain impact, tensile, and HDT specimens, according to ASTM D256, ASTM D638, and ASTM D648, respectively. The molding conditions of the specimens were: injection pressure = 1200 bar; Temperature profile: 230, 240, 240, 240, 245 °C; Mold temperature = 50 °C; Cooling time in the mold = 25 s; Hold pressure = 1000 bar. The specimens were stored in a silica desiccator and characterized only after 48 h of injection molding. Figure 13 shows a schematic representation of nanocomposite preparation.

#### 3.2.2. Characterization of Materials

The rheological studies were conducted in the oscillatory regime using a parallel-plate Anton Paar Physica MCR 301 rheometer (Anton Paar, Graz, Austria). Plates with a 25 mm diameter separated by 1 mm and angular frequency varying from 0.1 at 600 rad/s were used throughout the tests. The experiments were performed under an air atmosphere employing a temperature of 230 °C. 1% strain within the linear viscoelasticity region was adopted. Samples from impact test specimens were taken for analysis.

The Izod impact strength test was conducted in a Ceast device, model Resil 5.5 J, operating with a 5.5 J hammer (Ceast, Torino, Italy). Tests were performed on notched specimens, according to ASTM D256, at room temperature (~24 °C). Seven specimens were tested to obtain an average result. The samples presented dimensions of 3.3 mm, 12.8 mm, and 64 mm in thickness, width, and length, respectively.

Tensile tests were performed on injected specimens, according to ASTM D638, using an Oswaldo Filizola BME universal testing machine (Oswaldo Filizola, São Paulo, Brazil), applying a loading speed of 50 mm/min and a load cell of 10 kN. The tests were conducted at room temperature for an average of five specimens. The dimensions of the specimens were 3.3 mm, 12.9 mm, and 165 mm in thickness, width, and length, respectively.

Thermogravimetry analysis was performed on simultaneous TA Instruments SDT-Q600 TGA/DSC/DTA equipment (TA Instruments, New Castle, DE, USA). The analysis was performed under a nitrogen atmosphere (gas flow of 50 mL/min) by heating 6 mg samples from 30 to 600 °C with a 10 °C/min heating rate.

Current-voltage measurements were performed on a Keithley 2182A nanovoltmeter with a real-time recording (Keithley Instruments, Cleveland, OH, USA). The measurements were made in the –7 V to 7 V range. The electrical conductivity (*σ*) was determined by combining Equations (2) and (3), as suggested in the literature [78]:

(2) $$\rho =\frac{RLZ}{l}$$

(3) $$\sigma =\frac{1}{\rho \;}$$

where: ρ = electrical resistivity; *R* = electrical resistance; *L* = sample width; *Z* = sample thickness; *l* = distance between the two contact points in the sample.

Shielding effectiveness against electromagnetic interference was performed in a rectangular waveguide coupled to an Agilent Co model N5230C/PNA-L network analyzer (Agilent Technologies, Santa Clara, CA, USA). The shielding effect was evaluated with radiation in the 8.2–12.4 GHz frequency range. For measurements, samples were compression molded using 8 tons for 4 min to achieve a thickness of 1 mm.

Scanning electron microscopy (SEM) was performed on the fracture surface of impact-tested specimens (TESCAN, Brun, Tchéquia). A VEGAN TESCAN 3 scanning electron microscope working with 30 kV voltage and under a high vacuum was employed to examine the fracture surfaces of gold sputtered samples.

## 4. Conclusions

Toughened Nylon 6/ABS nanocomposites were prepared for potential application in antistatic and shielding against electromagnetic interference. The Nylon 6/ABS/ABS-g-MA blend showed good impact performance, evidencing the ABS-g-MA effectiveness as a reactive compatibilizing agent for Nylon 6/ABS systems. In light of this, the Nylon 6/ABS/ABS-g-MA system was suitable for nanocomposite preparation with MWCNT to create new materials with tailored properties for the electrical industry. MWCNT addition to the Nylon 6/ABS/ABS-g-MA mixture optimized the electrical conductivity and led to conductive nanocomposites. Moreover, the nanocomposites sustained impact strength and elastic modulus at high levels, acceptable for the production of nanocomposites with enhanced mechanical and electrical properties. For antistatic packaging purposes, incorporating 1 phr of carbon nanotubes was enough to generate high flexibility, impact strength, and electromagnetic radiation attenuating power. The highest electromagnetic shielding efficiency was attained for nanocomposites with 5 phr MWCNT, reaching an attenuation degree of about –10.5 dB at 9 GHz and –8.2 dB in the 11 to 12 GHz frequency range. The development of Nylon 6/ABS/ABS-g-MA/MWCNT nanocomposites holds technological potential due to their good properties and versatility. These make it possible to control the characteristics exhibited by these nanocomposites, allowing them to be molded to meet the required applications. Additionally, nanocomposites are lightweight, corrosion-resistant, cheaper, and more convenient for miniaturized parts than metals. The ongoing trend is to expand research on engineered nanocomposites using hybrid blends of nanofillers to achieve advanced multifunctional materials.

## Figures and Tables

**Table 1:** Mechanical properties of Nylon 6, blends, and nanocomposites. IS = impact strength; E = elastic modulus; TS = Tensile strength at yielding; EB = elongation at break; N = Nylon 6.

Samples	IS (J/m)	E (GPa)	TS (MPa)	EB (%)
Nylon 6 (N)	43.0 ± 2.0	2.95 ± 0.1	67.4 ± 1.1	36.7 ± 5.2
N/ABS	37.4 ± 2.2	2.21 ± 0.05	47.3 ± 0.5	4.8 ± 1.6
N/ABS/ABS-g-MA	181.9 ± 4.5	2.38 ± 0.06	42.1 ± 0.9	138.3 ± 9.8
N/ABS/ABS-g-MA/MWCNT (1 phr)	126.4 ± 5.0	2.41 ± 0.08	43.3 ± 0.7	94.1 ± 6.2
N/ABS/ABS-g-MA/MWCNT (3 phr)	96.5 ± 4.7	2.52 ± 0.04	43.4 ± 1.0	23.4 ± 2.3
N/ABS/ABS-g-MA/MWCNT (5 phr)	82.4 ± 5.0	2.63 ± 0.07	43.8 ± 0.5	5.3 ± 1.7

**Table 2:** Thermal stability results for pure polymers, blends, and nanocomposites. N = Nylon 6.

Samples	T_0.1_ (°C)	T_0.5_ (°C)
Nylon 6 (N)	384.6	446.1
ABS	396.2	427.9
ABS-MA	376.1	426.1
N/ABS	394.8	436.1
N/ABS/ABS-g-MA	391.5	439.6
N/ABS/ABS-g-MA/MWCNT (1 phr)	397.3	444.9
N/ABS/ABS-g-MA/MWCNT (3 phr)	398.4	445.6
N/ABS/ABS-g-MA/MWCNT (5 phr)	399.1	445.5

**Table 3:** Formulations of the processed nanocomposites. Part per hundred of resin (phr); N = Nylon 6.

Samples	Nylon 6 (%)	ABS (%)	ABS-g-MA (%)	MWCNT (phr)
Nylon 6 (N)	100	-	-	-
N/ABS	60	40	-	-
N/ABS/ABS-g-MA	60	30	10	-
N/ABS/ABS-g-MA/MWCNT	60	30	10	1
N/ABS/ABS-g-MA/MWCNT	60	30	10	3
N/ABS/ABS-g-MA/MWCNT	60	30	10	5
